# Dissolution Profiles of Immediate Release Products of Various Drugs in Biorelevant Bicarbonate Buffer: Comparison with Compendial Phosphate Buffer

**DOI:** 10.1007/s11095-024-03701-6

**Published:** 2024-04-23

**Authors:** Nanami Okamoto, Masaki Higashino, Hibiki Yamamoto, Kiyohiko Sugano

**Affiliations:** 1 Molecular Pharmaceutics Lab., College of Pharmaceutical Sciences, Ritsumeikan University, 1-1-1, Noji-Higashi, Kusatsu, Shiga 525-8577 Japan; 2 Research & Development Division, Towa Pharmaceutical Co., Ltd., 2-5-15, Hiyoshi-Cho, Moriguchi, Osaka 570-0081 Japan

**Keywords:** bicarbonate, biorelevant, dissolution, salt, supersaturation

## Abstract

**Purpose:**

The purpose of this study was to clarify the extent to which the dissolution profiles of immediate release (IR) products of various drugs differ between biorelevant bicarbonate buffer (BCB) and compendial phosphate buffer (PPB).

**Methods:**

The dissolution profiles of the IR products of fifteen poorly soluble ionizable drugs were measured in BCB and PPB. BCB was set to be relevant to the small intestine (pH 6.8, 10 mM). The pH was maintained using the floating lid method. The Japanese pharmacopeia second fluid (JP2, 25 mM phosphate buffer, nominal pH 6.8) was used as compendial PPB. The compendial paddle apparatus was used for the dissolution tests (500 mL, 50 rpm, 37°C).

**Results:**

In 11/15 cases, a difference in dissolved% (< 0.8 or > 1.25-fold) was observed at a time point. In 4/15 cases, the ratio of the area under the dissolution curve was not equivalent (< 0.8 or > 1.25-fold). In the cases of free-form drugs, the dissolution rate tended to be slower in BCB than in JP2. In the case of salt-form drugs, a marked difference was observed for the cases that showed supersaturation. However, no trend was observed in the differences.

**Conclusions:**

Many IR products showed differences in the dissolution profiles between biorelevant BCB and compendial PPB. With the floating lid method, BCB is as simple and easy to use as PPB. Biorelevant BCB is recommended for dissolution testing.

**Supplementary Information:**

The online version contains supplementary material available at 10.1007/s11095-024-03701-6.

## Introduction

Dissolution tests have been widely used to assess the bioavailability (BA) and bioequivalence (BE) of orally administered drugs in drug discovery, development, and manufacturing. Recently, biorelevant dissolution media have been intensively investigated to improve the BA/BE predictability of dissolution tests [1–6]. Biorelevant dissolution media should mimic gastrointestinal fluids as accurately as possible. The intestinal pH value is maintained by bicarbonate buffer (BCB) [7, 8]. Therefore, BCB should be used for biorelevant dissolution media. BCB maintains the pH value by the following chemical equilibrium.

1 $${HCO}_{3}^{-}+{H}^{+}\rightleftarrows {H}_{2}{CO}_{3}\rightleftarrows {H}_{2}O+{CO}_{2}$$

The reaction rate of CO_2_ hydration is significantly slower than that of H_2_CO_3_ dehydration. This unique property of BCB affects the dissolution rates of drug substances and products [9].

However, phosphate buffer (PPB) has been used for many years for practical reasons. When BCB is exposed to air, the pH value rapidly increases as CO_2_ volatilizes from the solution. A CO_2_ gas supply has been used to compensate for the loss of CO_2_ during dissolution testing [10–12]. However, this requires specialized equipment such as a CO_2_ gas cylinder, a gas regulator, and a pH monitor. In addition, the use of surfactants with gas bubbling causes foaming [13]. Furthermore, gas bubbling can have an artificial effect on the precipitation of a drug (manuscript submitted). To overcome these challenges, we recently developed the floating lid method [14]. In this method, a floating lid is placed on the surface of a BCB solution to prevent the loss of CO_2_. By using a floating lid, the pH increase can be kept below 0.1 pH units for several hours. The floating-lid method is simple, low-cost, robust, and easy to operate. It has already been applied to various experimental conditions [15, 16].

Previously, the dissolution rate of several active pharmaceutical ingredients (API) has been investigated in BCB [9, 17, 18]. For example, ibuprofen was reported to show a slower dissolution profile in BCB than in PPB [9]. In the case of salt form APIs, BCB and PPB differently affected the precipitation of its corresponding free forms at the dissolving particle surface [18]. However, the number of tested drugs was limited. In addition, raw drug substances have been used in these studies. Therefore, it has been unclear to what extent BCB affects the dissolution profiles of immediate-release (IR) products of various drugs.

**Fig. 1:**
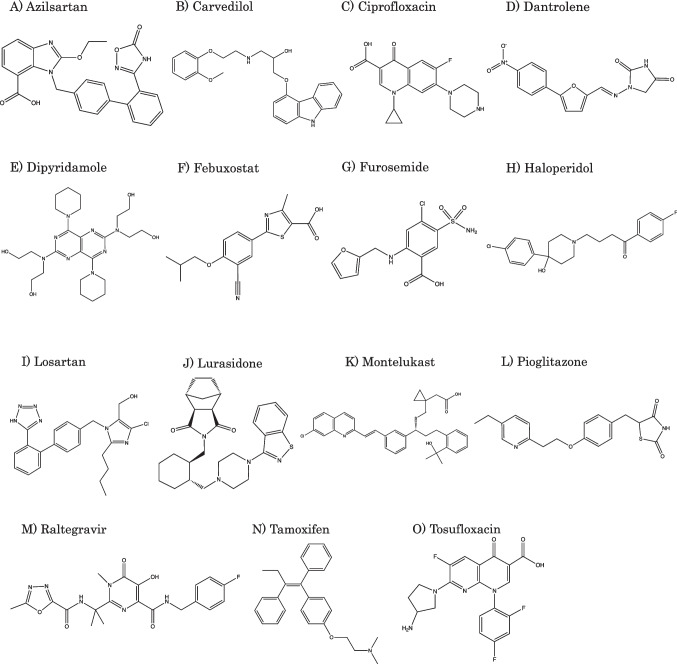
Chemical structures of model drugs.

The purpose of the present study was to clarify the extent to which the dissolution profiles of IR products of various drugs differ between biorelevant BCB and compendial PPB. In this study, the dissolution profiles of IR products of 15 drugs were determined in compendial PPB and biorelevant BCB (Fig. 1, Tables I and II). This study focused on poorly soluble ionizable drugs because the choice of dissolution media is critically important for such drugs (3 free acids, 3 free bases, 4 acid salts, 3 base salts, and 2 zwitterion salts). The Japanese pharmacopeia second fluid (JP2, phosphate buffer, 25 mM, nominal pH 6.8) [19] was used as a compendial PPB. The pH value of BCB was aligned with the nominal pH of JP2. The bicarbonate concentration and ionic strength (*I*) were set to be relevant to the physiological condition (10 mM and *I* = 0.14 M adjusted by NaCl).

**Table I:** Physicochemical Properties of Model Drugs ^a^

Drug	p*K*_*a*_	Intrinsic solubility (µg/mL)
Azilsartan	3.2 (A), 5.2 (A) (NA^b^)	2.46 (37°C)
Carvedilol	8.06 (B) (25°C, *I* = 0.15 M)	1.1 (37°C)
Ciprofloxacin HCl H_2_O	8.63 (B), 6.15 (A) (24°C, *I* = 0.16 M)	170 (40°C) ^c^
Dantrolene Na H_2_O	7.49 (A) (22°C, *I* = 0.1 M)	0.85 (23°C)
Dipyridamole	6.2 (B) (25°C, *I* = 0.15 M),	1.6 (22.5°C)
Febuxostat	3.59 (A) (NA)	0.94 (37°C)
Furosemide	3.53 (A) (37°C, *I* = 0.15 M)	5.9 (25°C)
Haloperidol	8.29 (B) (37°C, *I* = 0.15 M)	0.57 to 2.5 (23 to 37℃)
Losartan K	4.25 (A) (25°C, *I* = 0.15 M)	393 (NA) ^d^
Lurasidone HCl	7.6 (B) (NA)	0.026 (20°C) ^d^
Montelukast Na	2.8 (A), 5.7 (A) (25°C, *I* = 0.05 M)	0.2 (25°C)
Pioglitazone HCl	5.63 (B), 6.63 (A) (25°C, *I* = 0.15 M)	0.25 (25°C) ^d^
Raltegravir K	6.7 (A) (25°C)	22 (37°C)
Tamoxifen citrate	8.45 (B) (37°C, *I* = 0.18 M)	0.010 (25°C)
Tosufloxacin tosylate H_2_O	5.8 (A), 8.7(B) (NA)	2.1 (37°C) ^c^

a The physicochemical properties are mainly taken from ref. [20] and the drug product information. The list of references is available as Supplemental Information Table S1

b Temperature and ionic strength data were not available

c A zwitterion drug. Solubility near the isoelectric point

d Calculated from p*K*_*a*_ and a solubility value at a pH

**Table II:** Formulations and Manufactures

Drug	Formulations	Manufacturer
Azilsartan	AZILVA Tablets 40 mg	Takeda Pharmaceutical Company Limited
Carvedilol	ARTIST Tablets 2.5 mg	DAIICHI SANKYO COMPANY, LIMITED
Ciprofloxacin HCl H_2_O	Ciprofloxacin Tablets 200 mg 「SW」	Sawai Pharmaceutical Co., Ltd
Dantrolene Na H_2_O	Dantrium Capsules 25 mg	OrphanPacific, Inc
Dipyridamole	Persantin Tablets 100 mg	Medical Parkland K.K
Febuxostat	Feburic Tablets 40 mg	TEIJIN PHARMA LIMITED
Furosemide	Rasix Tablets 20 mg	Sanofi K.K
Haloperidol	Serenace Tablets 3 mg	Sumitomo Pharma Co., Ltd
Losartan K	Losartan Potassium Tablets 25 mg 「SW」	Sawai Pharmaceutical Co., Ltd
Lurasidone HCl	Latuda Tablets 60 mg	Sumitomo Pharma Co., Ltd
Montelukast Na	Montelukast Tab 10 mg「EE」	Nichi-Iko Pharmaceutical Co., Ltd
Pioglitazone HCl	ACTOS Tablets 30 mg	Teva Takeda Pharma Ltd
Raltegravir K	ISENTRESS Tablets 400 mg	Merck & Co., Inc
Tamoxifen citrate	Tamoxifen Citrate Tablets 20 mg 「SW」	Sawai Pharmaceutical Co., Ltd
Tosufloxacin tosylate H_2_O	Tosufloxacin Tosylate Hydrate Tablets 150 mg 「SW」	Sawai Pharmaceutical Co., Ltd

## Materials and Methods

### Materials

Azilsartan, carvedilol, ciprofloxacin hydrochloride hydrate, dipyridamole, dantrolene sodium hydrate, furosemide, febuxostat, haloperidol, losartan potassium, lurasidone, montelukast sodium hydrate, and pioglitazone hydrochloride were purchased from Tokyo Chemical Industry Co., Ltd. Tamoxifen citrate and tosufloxacin tosylate hydrate were purchased from FUJIFILM Wako Pure Chemical Corporation. Raltegravir was extracted from the tablet. The manufacturers of the IR products are summarized in Table II.

### Methods

A compendial paddle dissolution apparatus (NTR-6200A; Toyama Sangyo Co., Ltd., Osaka, Japan) was used for the dissolution test. The pH value was measured using the 9615S-10D Standard ToupH electrode (HORIBA Advanced Techno, Co., Ltd., Kyoto, Japan). The floating lid method was used to maintain the pH value of BCB (pH 6.8, 10 mM bicarbonate, *I* = 0.14 M (adjusted by NaCl)). The floating lid (foamed styrol, thickness: 5 mm) was designed to cover more than 95% of the surface area of a buffer solution [14].

A NaHCO_3_ solution (490 ml, 10.2 mM, NaCl 0.13 M, prewarmed at 37°C in a container for at least 30 min) was added to each vessel. The temperature was maintained at 37°C. The paddle rotation speed was set to 50 rpm. An HCl solution (10 ml, 0.113 M) was added to adjust the pH value to pH 6.8 (this HCl concentration (0.00226 M after dilution) was experimentally determined to give pH 6.8 after adding to the NaHCO_3_ solution). The solution surface was covered by a floating lid. As a compendial phosphate buffer solution, the Japanese pharmacopeia second fluid (JP2, 25 mM, phosphate buffer, nominally pH 6.8) was used. The actual pH value of JP2 was reported to be pH 6.9 [19]. The other conditions were the same as the BCB buffer, including the use of the floating lid.

One tablet or capsule was added to each vessel (except for tosufloxacin (two tablets)). At specified time intervals, a small volume of samples (1.0 ml) was withdrawn and immediately filtered (hydrophilic PVDF, φ = 4 mm, pore size: 0.22 µm, Merck). The first few droplets were discarded to avoid filter adsorption. The filtrate was diluted with an appropriate medium, and the concentrations of the drugs were measured by UV absorbance (except for lurasidone) (UV-1850, Shimazu Corporation, Kyoto, Japan, or SH-9500lab, CORONA ELECTRIC, Ibaraki, Japan). The detection wavelength, the concentration range, the number of data points, and the determination coefficient (*r*^*2*^) of standard curves are summarized in Supplemental Material Table S2. The absence of UV interference from the excipients was confirmed by comparing the UV spectrum of a pure API and its product. The concentration of lurasidone was quantified by HPLC (Shimazu Prominence LC-20 series; Column: Zorbax Eclipse Plus C18, 2.1 × 50 mm, 3.5 μm; mobile phase: acetonitrile/ 0.1% trifluoroacetic acid (40: 60); flow rate: 0.6 mL/min; temperature: 40°C; detection wavelength: 320 nm; injection volume: 10 μL). The dissolution test was performed in triplicate. The area under the dissolution curve (AUDC) was calculated by the trapezoidal method.

The *β* values before adding a drug were calculated using the p*K*_*a*_ values at a similar ionic strength reported in the literature (BCB: p*K*_*a*_=6.05 (for *I*=0.15, at 37°C), PPB: p*K*_*a*_=6.9 (for *I*=0.05, at 37°C)) [20–22].

## Results

**Fig. 2:**
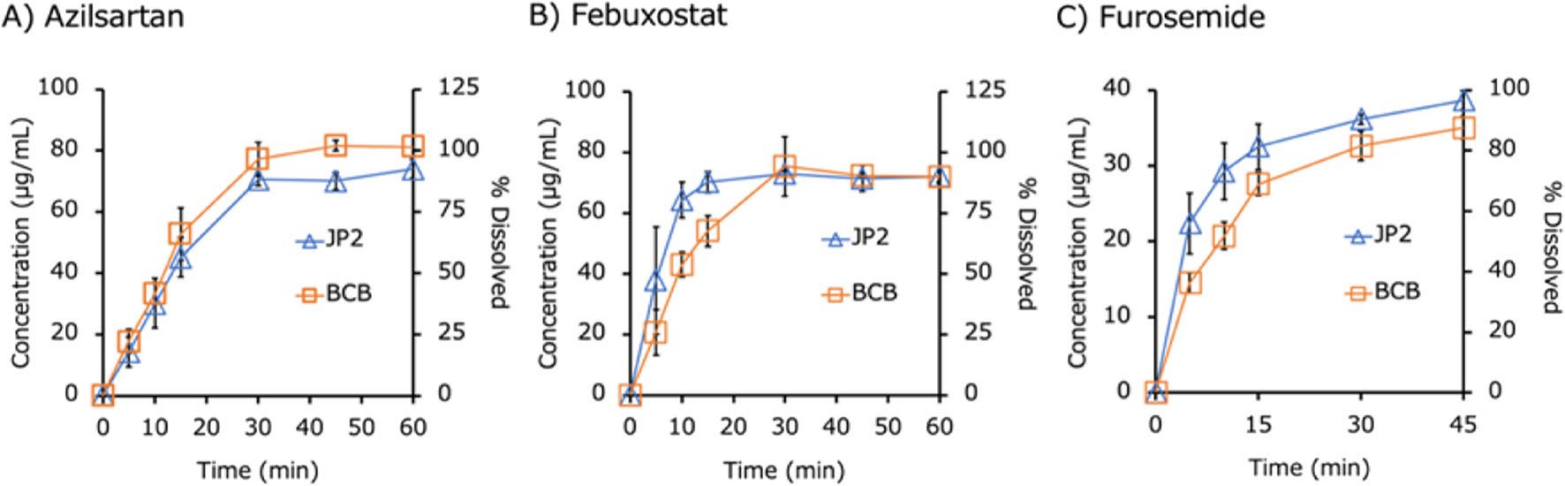
Dissolution profiles of IR products of free acids in biorelevant BCB and compendial PPB (JP2) (mean ± S.D., N = 3). (**A**) Azilsartan, (**B**) febuxostat, and (**C**) furosemide.

The dissolution profiles of the IR products are shown in Figs. 2 (free acids), 3 (free bases), 4 (acid salts), 5 (base salts), and 6 (others). The drug concentration was reported as a free form. The initial pH values of BCB and PPB were pH 6.80 ± 0.05 (mean ± S.D., N = 45) and 6.96 ± 0.02 (mean ± S.D., N = 45) (Supplemental Information Table S3). The final pH values of BCB were in the range of 6.80 to 7.00 except for dantrolene Na H_2_O (pH 7.10 ± 0.00) (mean ± S.D., N = 3 (the same hereinafter)), tamoxifen citrate (pH 7.17 ± 0.03), lurasidone HCl (pH 7.22 ± 0.05), raltegravir K (pH 7.11 ± 0.01), ciprofloxacin HCl (pH 6.76 ± 0.03), and tosufloxacin tosylate H_2_O (pH 6.60 ± 0.05). The final pH values of PPB were in the range of 6.90 to 7.10 except for tosufloxacin tosylate H_2_O (pH 6.81 ± 0.02). The ratio of AUDC (AUDCr), the *f*2 values, and the maximum or minimum ratio of dissolved% at a time point (*D*%*ratio*) are summarized in Table III. In 4/15 cases, AUDC was not equivalent (AUDCr < 0.8 or > 1.25-fold). When comparing *D*%*ratio* at each time point, a difference of < 0.8 or > 1.25-fold was observed in 11/15 cases. The *f*2 value was not used because it is usually valid only for complete dissolution cases [23–25].

**Fig. 3:**
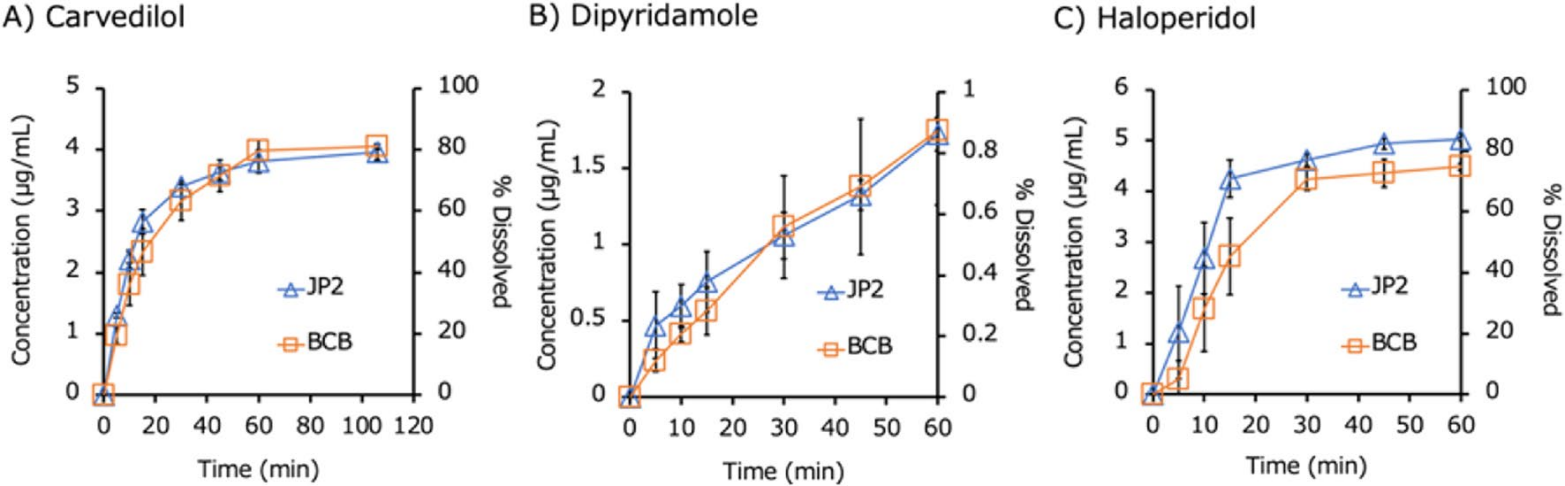
Dissolution profiles of IR products of free bases in biorelevant BCB and compendial PPB (JP2) (mean ± S.D., N = 3). (**A**) Carvedilol, (**B**) dipyridamole, and (**C**) haloperidol.

**Fig. 4:**
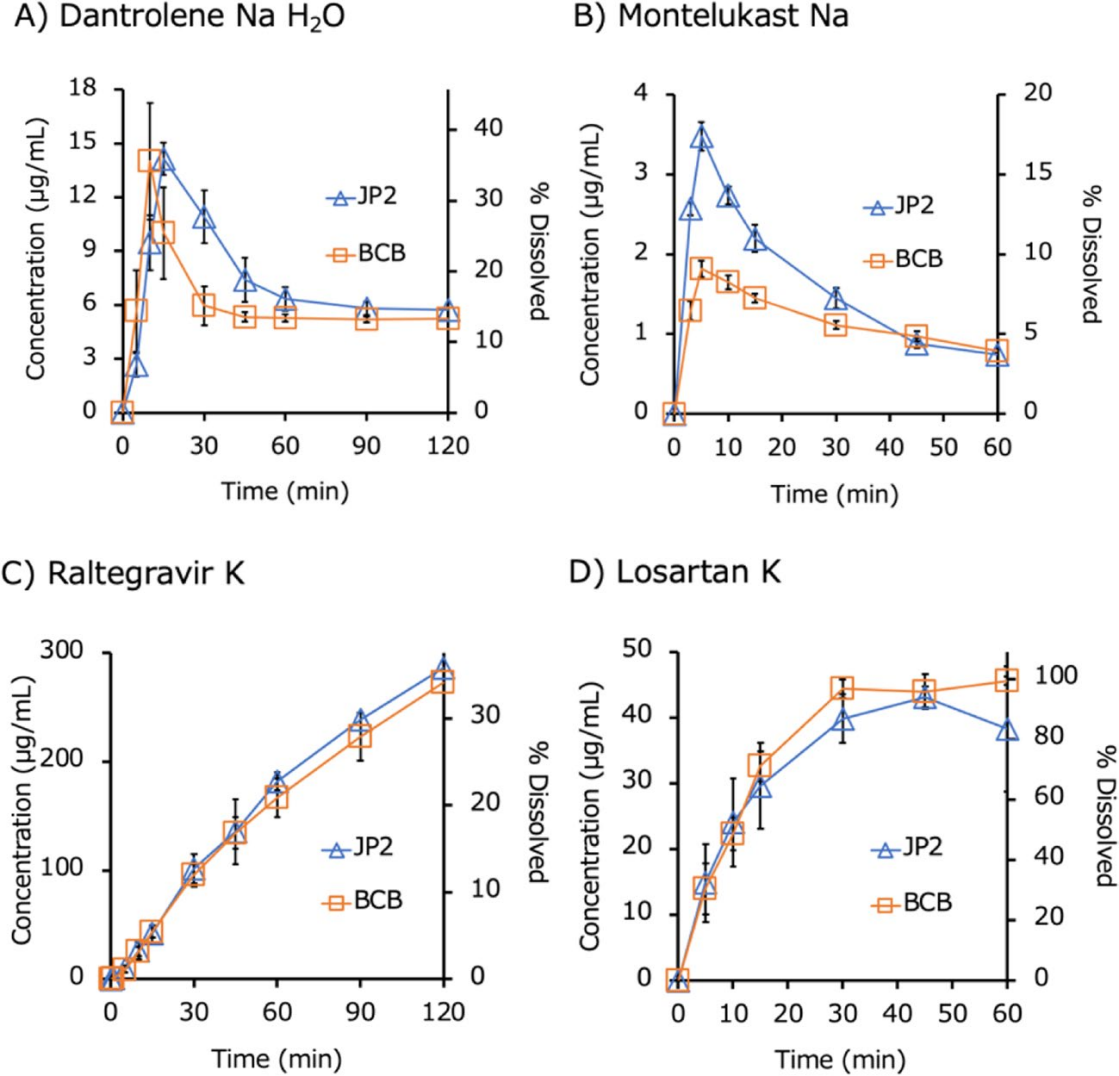
Dissolution profiles of IR products of acid salts in biorelevant BCB and compendial PPB (JP2) (mean ± S.D., N = 3). (**A**) Dantrolene Na H_2_O, (**B**) montelukast Na, (**C**) raltegravir K, and (**D**) losartan K.

**Fig. 5:**
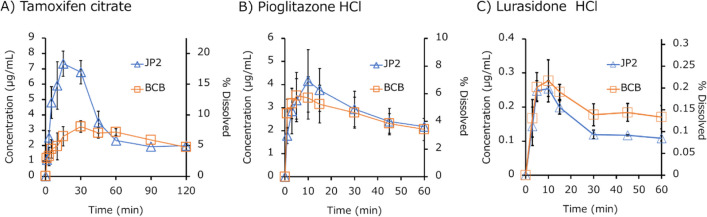
Dissolution profiles of IR products of base salts in biorelevant BCB and compendial PPB (JP2) (mean ± S.D., N = 3). (**A**) Tamoxifen citrate, (**B**) pioglitazone HCl, and (**C**) lurasidone HCl.

**Fig. 6:**
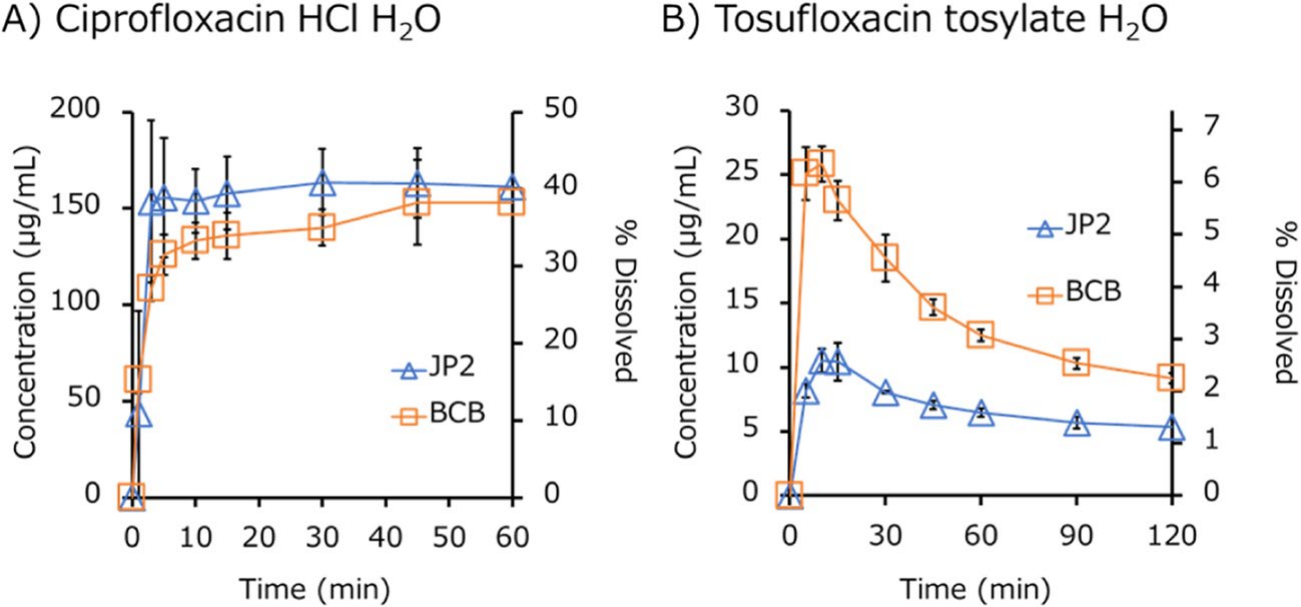
Dissolution profiles of IR products pf zwitterion salt in biorelevant BCB and compendial PPB (JP2) (mean ± S.D., N = 3). (**A**) Ciprofloxacin HCl H_2_O, and (**B**) tosufloxacin tosylate H_2_O.

**Table III:** AUDC Ratio and *D% Ratio*

API	Drug amount (mg)	AUDCr^a^	*D*% ratio ^a, b^
Azilsartan	40	1.131	1.16 (45 min)
Carvedilol	2.5	0.985	0.825 (15 min)
Ciprofloxacin HCl H_2_O	200	0.887	0.707 (3 min)
Dantrolene Na H_2_O	25	0.820	0.545 (30 min)
Dipyridamole	100	0.966	0.511 (5 min)
Febuxostat	40	0.922	0.670 (10 min)
Furosemide	20	0.851	0.707 (10 min)
Haloperidol	3	0.821	0.639 (15 min)
Losartan K	25	1.075	1.19 (60 min)
Lurasidone HCl	60	1.339	1.57 (45 min)
Montelukast Na	10	0.733	0.523 (5 min)
Pioglitazone HCl	30	0.934	1.54 (1 min)
Raltegravir K	400	0.944	0.934 (90 min)
Tamoxifen citrate	20	0.715	0.356 (15 min)
Tosufloxacin tosylate H_2_O	300	2.084	2.46 (10 min)

a BCB/ JP2

b Maximum or minimum *D% ratio* in the dissolution profile. The time point is shown in parentheses

The dissolution rate of free-form drugs tended to be slower in BCB than in PPB except for azilsartan. In the case of salt-form drugs, marked differences were observed in initial dissolution and supersaturation profiles in many cases such as dantrolene Na H_2_O, montelukast Na H_2_O, tamoxifen citrate, and tosufloxacin tosylate H_2_O. However, no trend was observed in the differences. The difference in the dissolved drug concentrations became smaller after 60 min except for lurasidone HCl.

## Discussion

This study compared for the first time the dissolution profiles of IR products of a wide range of poorly soluble ionizable drugs in biorelevant BCB and compendial PPB (JP2). The results demonstrated that a significant portion of IR products showed marked differences in the initial dissolution and supersaturation profiles between biorelevant BCB and JP2.

Theoretically, the equilibrium pH and solubility of a drug should be similar for BCB and PPB when the buffer capacity is sufficient. In this study, the pH value of the bulk phase after dissolution testing and the dissolved drug concentration after 60 min were similar between BCB and JP2. These experimental results were in good agreement with the theory. However, due to the slow hydration rate of CO_2_, the neutralization rate of BCB is much slower than that of PPB. The differences in the initial dissolution and supersaturation profile would be attributed to the pH value at the surface of drug particles that can be affected by the neutralization rate of buffer species.

In the cases of free-form drugs, theoretically, the dissolution rate should be slower in BCB than in JP2 because the particle surface pH is more slowly neutralized by BCB [9, 26]. The result of this study was qualitatively in good agreement with the theory. Theoretical quantitative prediction of the particle surface pH of BCB requires information on the particle size of the drug substance, which is often not available for commercial products. Agitation conditions may also affect the particle surface pH because the effective p*K*_*a*_ of BCB is a function of the hydrodynamics [26]. The difference in particle surface pH between BCB and PPB can be more than 0.5 pH unit, resulting in a threefold or greater difference in dissolution rates [27, 28]. Theoretical quantitative predictions of surface pH and dissolution rate are important and should be investigated further in the future.

In the cases of salt-form drugs, free-form precipitation can occur either on the particle surface during particle dissolution or in the bulk phase after particle dissolution [29, 30], both of which are affected by the neutralization rate of buffer species [31]. Theoretically, the dissolution of salt particles should be faster in BCB than in JP2 because pH neutralization at the particle surface should be slower in BCB than in JP2. In addition, the bulk phase precipitation of a free form should be slower in BCB than in JP2 (manuscript submitted). Therefore, in theory, more significant supersaturation should be observed in BCB than in JP2. However, the results of this study were not simply predicted by the above-mentioned theory, suggesting that more complex mechanisms exist for the dissolution and precipitation of a salt-form drug [17, 18]. In addition, an IR product contains various excipients that potentially affect the precipitation of a drug.

In this study, the buffer capacity (*β*) was different between BCB and PPB (BCB: *β* = 3.0 mM/pH, PPB (JP2): *β* = 14 mM/pH) [20–22]. In addition, the ionic strength (*I*) was also different. In our previous study, even when *β* and *I* were aligned between BCB and PPB, the dissolution profiles of salt drugs were markedly different [18]. The buffer capacity of the compendial PPB of USP is twice greater than that of JP2 [19]. Therefore, a more significant difference could be observed between biorelevant BCB and USP PPB.

In the case of salt-form APIs, supersaturation was observed except for raltegravir K and ciprofloxacin HCl H_2_O. In the case of raltegravir K, visual observation of the dissolution test suggested that tablet disintegration was likely to be the rate-limiting process. Komasaka *et al*. previously reported that the dissolution rate of the raltegravir K tablet was affected by pre-exposure to an acidic pH environment due to its conversion to an insoluble free acid form [32]. Therefore, a pH shift process should be coupled with BCB for further investigation [16]. For ciprofloxacin HCl H_2_O, it was not clear why no supersaturation was observed in the dissolution profile.

In conclusion, a significant portion of IR products showed differences in the dissolution profiles between biorelevant BCB and compendial PPB, especially for salt-form drugs. With the floating lid method, BCB is as simple and easy to use as PPB. The advantages of BCB have been demonstrated for the evaluation of enteric-coated products [33, 34], sustained-release products [35], and amorphous solid dispersions [36]. The result of this study suggested that BCB is recommended as a first choice for biorelevant dissolution tests of IR products.

### Supplementary Information

Below is the link to the electronic supplementary material.

Supplementary file1 (DOCX 32.8 KB)

## Data Availability

The data that support the findings of this study are available on request from the corresponding author.
