# Supplementary material for: Dissolution Profiles of Immediate Release Products of Various Drugs in Biorelevant Bicarbonate Buffer: Comparison with Compendial Phosphate Buffer
Source: Pharm Res. 2024 Apr 23;41(5):959–66. doi: 10.1007/s11095-024-03701-6 (PMC11116250; doi:10.1007/s11095-024-03701-6)
Supplement: Supplementary file 1 — Supplementary file1 (DOCX 32.8 KB) [file 11095_2024_3701_MOESM1_ESM.docx]

Supplemental Information

Dissolution profiles of immediate release products of various drugs in biorelevant bicarbonate buffer: comparison with compendial phosphate buffer

Nanami Okamoto^1^, Masaki Higashino^1,2^, Hibiki Yamamoto^1^, Kiyohiko Sugano^1,*^

1. Molecular Pharmaceutics Lab., College of Pharmaceutical Sciences, Ritsumeikan University, 1-1-1, Noji-higashi, Kusatsu, Shiga 525-8577, Japan

2. Research & Development Division, Towa Pharmaceutical Co., Ltd, 2-5-15, Hiyoshi-cho, Moriguchi, Osaka 570-0081, Japan.

* Corresponding author. Tel.: +81-77-561-2773; E-mail address: suganok@fc.ritsumei.ac.jp (K. Sugano).

Table S1 References for physicochemical properties

| Drug | p*K_a_* | Intrinsic solubility |
| --- | --- | --- |
| Azilsartan | Drug product information | Lu, T., Sun, Y., Ding, D., Zhang, Q., Fan, R., He, Z., & Wang, J. (2017). Study on enhanced dissolution of azilsartan-loaded solid dispersion, prepared by combining wet milling and spray-drying technologies. *AAPS PharmSciTech*, *18*, 473-480. |
| Carvedilol | Alex, A. (2012) Absorption and Drug Development: Solubility, Permeability, and Charge State Wiley; 2nd edition. John Wiley & Sons, Inc. | Hsieh, Y. L., Ilevbare, G. A., Van Eerdenbrugh, B., Box, K. J., Sanchez-Felix, M. V., & Taylor, L. S. (2012). “pH-induced precipitation behavior of weakly basic compounds: determination of extent and duration of supersaturation using potentiometric titration and correlation to solid state properties” *Pharmaceutical research*, *29*, 2738-2753. |
| Ciprofloxacin HCl H_2_O | Alex, A. (2012) Absorption and Drug Development: Solubility, Permeability, and Charge State Wiley; 2nd edition. John Wiley & Sons, Inc. | Yu, X., Zipp, G. L., & Davidson III, G. R. (1994). The effect of temperature and pH on the solubility of quinolone compounds: estimation of heat of fusion. *Pharmaceutical research*, *11*, 522-527. |
| Dantrolene Na H_2_O | Livertoux, M. H., Jayyosi, Z., & Batt, A. M. (1988). Study of the physicochemical properties of aqueous dantrolene solutions by differential pulse polarography. *Talanta*, *35*(8), 613-619. | Wuis, E. W., Grutters, A. C. L. M., Vree, T. B., & Van Der Kleyn, E. (1982). Simultaneous determination of dantrolene and its metabolites, 5-hydroxydantrolene and nitro-reduced acetylated dantrolene (F 490), in plasma and urine of man and dog by high-performance liquid chromatography. *Journal of Chromatography B: Biomedical Sciences and Applications*, *231*(2), 401-409. |
| Dipyridamole | Bendels, S., Tsinman, O., Wagner, B., Lipp, D., Parrilla, I., Kansy, M., & Avdeef, A. (2006). PAMPA–excipient classification gradient map. Pharmaceutical research, 23, 2525-2535. | Bergström, C. A., Luthman, K., & Artursson, P. (2004). Accuracy of calculated pH-dependent aqueous drug solubility. *European journal of pharmaceutical sciences*, *22*(5), 387-398. |
| Febuxostat | Jagia, M., Daptardar, R., Patel, K., Bansal, A. K., & Patel, S. (2019). Role of structure, microenvironmental pH, and speciation to understand the formation and properties of febuxostat eutectics. *Molecular Pharmaceutics*, *16*(11), 4610-4620. | Sharma, O. P., Patel, V., & Mehta, T. (2016). Design of experiment approach in development of febuxostat nanocrystal: application of Soluplus® as stabilizer. Powder technology, 302, 396-405. |
| Furosemide | Alex, A. (2012) Absorption and Drug Development: Solubility, Permeability, and Charge State Wiley; 2nd edition. John Wiley & Sons, Inc. | Avdeef, A., Berger, C. M., & Brownell, C. (2000). pH-metric solubility. 2: correlation between the acid-base titration and the saturation shake-flask solubility-pH methods. Pharmaceutical research, 17, 85-89. |
| Haloperidol | Alex, A. (2012) Absorption and Drug Development: Solubility, Permeability, and Charge State Wiley; 2nd edition. John Wiley & Sons, Inc. | Li, S., Wong, S., Sethia, S., Almoazen, H., Joshi, Y. M., & Serajuddin, A. T. (2005). Investigation of solubility and dissolution of a free base and two different salt forms as a function of pH. *Pharmaceutical research*, *22*, 628-635.  Bergström, C. A., & Avdeef, A. (2019). Perspectives in solubility measurement and interpretation. ADMET and DMPK, 7(2), 88-105. |
| Losartan K | Alex, A. (2012) Absorption and Drug Development: Solubility, Permeability, and Charge State Wiley; 2nd edition. John Wiley & Sons, Inc. | Tran, T. H., Park, C., Kang, T., Park, Y. J., Oh, E., & Lee, B. J. (2015). Micromeritic properties and instrumental analysis of physical mixtures and solid dispersions with adsorbent containing losartan: Comparison of dissolution-differentiating factors. Powder Technology, 272, 269-275**.** |
| Lurasidone HCl | Center for Drug Evaluation and Research, U.S. Food and Drug Administration. Latuda (lurasidone hydrochloride) tablets. | Center for Drug Evaluation and Research, U.S. Food and Drug Administration. Latuda (lurasidone hydrochloride) tablets. |
| Montelukast Na H_2_O | Thibert, R., Mach, H., Clas, S. D., Meisner, D. R., & Vadas, E. B. (1996). Characterization of the self-association properties of a leukotriene D4 receptor antagonist, MK-0476. *International journal of pharmaceutics*, *134*(1-2), 59-70. | Thibert, R., Mach, H., Clas, S. D., Meisner, D. R., & Vadas, E. B. (1996). Characterization of the self-association properties of a leukotriene D4 receptor antagonist, MK-0476. *International journal of pharmaceutics*, *134*(1-2), 59-70. |
| Pioglitazone HCl | Schönherr, D., Wollatz, U., Haznar-Garbacz, D., Hanke, U., Box, K. J., Taylor, R., ... & Weitschies, W. (2015). Characterisation of selected active agents regarding pKa values, solubility concentrations and pH profiles by SiriusT3. *European Journal of Pharmaceutics and Biopharmaceutics*, *92*, 155-170. | Schönherr, D., Wollatz, U., Haznar-Garbacz, D., Hanke, U., Box, K. J., Taylor, R., ... & Weitschies, W. (2015). Characterisation of selected active agents regarding pKa values, solubility concentrations and pH profiles by SiriusT3. *European Journal of Pharmaceutics and Biopharmaceutics*, *92*, 155-170. |
| Raltegravir K | Moss, D. M., Siccardi, M., Murphy, M., Piperakis, M. M., Khoo, S. H., Back, D. J., & Owen, A. (2012). Divalent metals and pH alter raltegravir disposition in vitro. *Antimicrobial agents and chemotherapy*, *56*(6), 3020-3026. | Komasaka, T., & Dressman, J. (2021). Simulation of oral absorption from non-bioequivalent dosage forms of the salt of raltegravir, a poorly soluble acidic drug, using a physiologically based biopharmaceutical modeling (PBBM) approach. European Journal of Pharmaceutical Sciences, 157, 105630. |
| Tamoxifen citrate | Alex, A. (2012) Absorption and Drug Development: Solubility, Permeability, and Charge State Wiley; 2nd edition. John Wiley & Sons, Inc. | Alex, A. (2012) Absorption and Drug Development: Solubility, Permeability, and Charge State Wiley; 2nd edition. John Wiley & Sons, Inc. |
| Tosufloxacin tosylate H_2_O | Drug product information | Yamamoto, H., Shanker, R., & Sugano, K. (2023). Application of Population Balance Model to Simulate Precipitation of Weak Base and Zwitterionic Drugs in Gastrointestinal pH Environment. *Molecular Pharmaceutics*, *20*(4), 2266-2275. |

Table S2 Wavelength and standard curves for concentration measurement

| Drug | Wavelength (nm) | Concentration range (µg/mL) | r2 |
| --- | --- | --- | --- |
| Azilsartan | 250 | 2.019 - 24.23 (BCB)  1.604 - 25.67 (JP2) | 0.9989 (BCB) 0.9998 (JP2) |
| Carvedilol | 286 | 2.015 - 32.24 | 0.9994 (BCB) 0.9997 (JP2) |
| Ciprofloxacin HCl H_2_O | 328 | 1.177 – 37.67 | 0.9999 (BCB) 1.0000 (JP2) |
| Dantrolene Na H_2_O | 381 | 1.195 - 21.85 | 1.0000 (BCB) 0.9999 (JP2) |
| Dipyridamole | 280 | 0.3139 - 10.05 | 0.9997 (BCB) 0.9989 (JP2) |
| Febuxostat | 314 | 1.148 - 18.38 | 0.9999 (BCB) 0.9999 (JP2) |
| Furosemide | 273 | 1.530 - 24.48 | 0.9998 (BCB) 0.9987 (JP2) |
| Haloperidol | 250 | 1.179 - 9.433 | 0.9986 (BCB)  0.9998 (JP2) |
| Losartan K | 250 | 4.784 - 38.27 | 0.9994 (BCB) 0.9997 (JP2) |
| Lurasidone HCl | - ^a^ | 0.08955 - 1.433 (BCB)  0.08089 - 10.35 (JP2) | 0.9999 (BCB) 1.0000 (JP2) |
| Montelukast Na H_2_O | 360 | 0.8981 - 14.37 | 0.9998 (BCB) 0.9999 (JP2) |
| Pioglitazone HCl | 270 | 0.7394 - 11.83 | 0.9997 (BCB) 0.9999 (JP2) |
| Raltegravir K | 350 | 3.125 - 100 | 1.0000 (BCB) 1.0000 (JP2) |
| Tamoxifen citrate | 280 | 0.7544 - 12.07 | 0.9931 (BCB) 0.9960 (JP2) |
| Tosufloxacin tosylate H_2_O | 346 | 2.829 - 11.32 | 0.9911 (BCB) 0.9861 (JP2) |

a Determined by HPLC (see text)

Table S3 Initial and final pH (mean ± S.D., N = 3)

|  | BCB |  | JP2 |  |
| --- | --- | --- | --- | --- |
| Drug | Initial pH | Final pH | Initial pH | Final pH |
| Azilsartan | 6.80 ± 0.01 | 6.81 ± 0.01 | 6.94 ± 0.00 | 6.93 ± 0.01 |
| Carvedilol | 6.78 ± 0.03 | 6.96 ± 0.01 | 6.98 ± 0.01 | 7.00 ± 0.00 |
| Ciprofloxacin HCl H_2_O | 6.76 ± 0.01 | 6.76 ± 0.03 | 6.96 ± 0.01 | 6.91 ± 0.00 |
| Dantrolene Na H_2_O | 6.77 ± 0.00 | 7.10 ± 0.00 | 6.96 ± 0.00 | 7.09 ± 0.00 |
| Dipyridamole | 6.80 ± 0.03 | 6.99 ± 0.07 | 6.96 ± 0.00 | 6.97 ± 0.00 |
| Febuxostat | 6.84 ± 0.03 | 6.99 ± 0.00 | 6.97 ± 0.01 | 6.96 ± 0.00 |
| Furosemide | 6.79 ± 0.01 | 6.88 ± 0.07 | 6.98 ± 0.00 | 6.97 ± 0.01 |
| Haloperidol | 6.81 ± 0.01 | 6.98 ± 0.01 | 6.97 ± 0.01 | 7.03 ± 0.01 |
| Losartan K | 6.79 ± 0.03 | 6.87 ± 0.00 | 6.97 ± 0.01 | 6.99 ± 0.00 |
| Lurasidone HCl | 6.84 ± 0.01 | 7.22 ± 0.05 | 6.94 ± 0.02 | 6.96 ± 0.00 |
| Montelukast Na H_2_O | 6.76 ± 0.01 | 6.94 ± 0.06 | 6.95 ± 0.01 | 7.01 ± 0.02 |
| Pioglitazone HCl | 6.80 ± 0.02 | 6.94 ± 0.00 | 6.94 ± 0.00 | 7.01 ± 0.09 |
| Raltegravir K | 6.93 ± 0.09 | 7.11 ± 0.01 | 6.98 ± 0.00 | 7.03 ± 0.00 |
| Tamoxifen citrate | 6.84 ± 0.01 | 7.17 ± 0.03 | 6.97 ± 0.01 | 6.97 ± 0.01 |
| Tosufloxacin tosylate H_2_O | 6.73 ± 0.04 | 6.60 ± 0.05 | 6.99 ± 0.01 | 6.81 ± 0.02 |
